# Generation of targeted mutant rice using a CRISPR‐Cpf1 system

**DOI:** 10.1111/pbi.12669

**Published:** 2017-02-19

**Authors:** Rongfang Xu, Ruiying Qin, Hao Li, Dongdong Li, Li Li, Pengcheng Wei, Jianbo Yang

**Affiliations:** ^1^ Key Laboratory of Rice Genetic Breeding of Anhui ProvinceRice Research InstituteAnhui Academy of Agricultural SciencesHefeiChina

**Keywords:** genome editing, Cpf1, CRISPR, rice

## Abstract

CRISPR‐Cpf1 is a newly identified CRISPR‐Cas system, and Cpf1 was recently engineered as a molecular tool for targeted genome editing in mammalian cells. To test whether the engineered CRISPR‐Cpf1 system could induce the production of rice mutants, we selected two genome targets in the *OsPDS* and *OsBEL* genes. Our results show that both targets could be efficiently mutated in transgenic rice plants using CRISPR‐Cpf1. We found that pre‐crRNAs with a full‐length direct repeat sequence exhibited considerably increased efficiencies compared with mature crRNAs. In addition, the specificity and transmission of the mutation were investigated, and the behaviours of crRNA‐Cpf1‐induced plant targeted genome mutagenesis were assessed. Taken together, our results indicate that CRISPR‐Cpf1 expression via stable transformation can efficiently generate specific and heritable targeted mutations in rice and thereby constitutes a novel and important approach to specific and precise plant genome editing.

## Introduction

The CRISPR‐Cas9 system has emerged as a precise and versatile technique for genome editing, and mounting research demonstrates that efficient editing could be induced by this system in various plant organisms, such as Arabidopsis, rice, wheat, soya bean, maize, tobacco and tomato (Belhaj *et al*., 2015; Bortesi and Fischer, 2015; Liu *et al*., 2016). Furthermore, the development and customization of plant CRISPR/Cas9 toolboxes or platforms result in more powerful molecular tools for fundamental research and crop breeding (Lowder *et al*., 2015; Ma *et al*., 2015; Mao *et al*., 2016; Tang *et al*., 2016; Wang *et al*., 2015; Xie *et al*., 2015; Xing *et al*., 2014). Rice is the most frequently used crop species for testing and applying CRISPR‐Cas9 tools. Several agronomic traits of rice, including yield, fertility, architecture, biotic and abiotic stress response, and herbicide tolerance, have been successfully modified by targeted mutations in one or multiple major genes (Ikeda *et al*., 2016; Li *et al*., 2016a,b,c; Osakabe *et al*., 2016; Sun *et al*., 2016; Wang *et al*., 2016; Xu *et al*., 2014, 2016; Zhou *et al*., 2015).

The recently identified type V CRISPR‐Cas system CRISPR‐Cpf1 was found to mediate targeted genome modification in mammalian cells (Hur *et al*., 2016; Kim *et al*., 2016; Zetsche *et al*., 2015). Cpf1 is an RNA‐guided endonuclease. Both crRNA and tracrRNA are required for Cas9‐mediated targeting. In contrast, a single ~44‐nucleotide (nt) crRNA with a 5′‐located direct repeat sequence and a spacer sequence that complements the target are sufficient to guide the DNA cleavage of Cpf1 (Zetsche *et al*., 2015). Moreover, Cpf1 recognizes the thymidine‐rich protospacer‐adjacent motif (PAM) and makes staggered cuts distal to the PAM site (Fonfara *et al*., 2016). Here, we demonstrated that efficient targeted mutagenesis can be achieved through the stable expression of certain types of crRNA and *Cpf1* in rice (*Oryza sativa*), a major crop.

## Results and discussion

**Figure 1:**
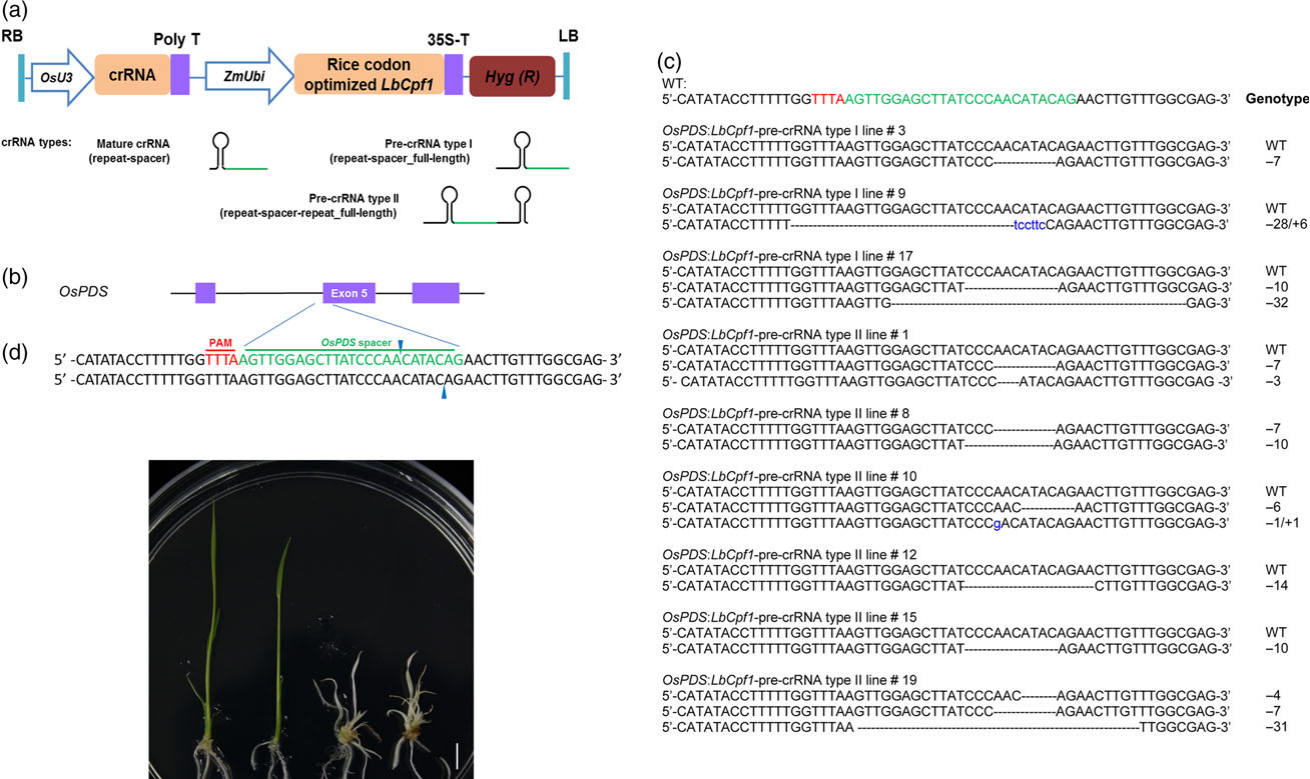
CRISPR‐Cpf1 induces targeted genome mutagenesis in rice through genetic transformation. (a) Schematic illustration of the T‐DNA region of the engineered CRISPR‐Cpf1 binary vector. Three types of crRNAs were used to form different crRNA‐Cpf1 combinations. The spacer and repeat regions of the crRNAs are marked in green and black, respectively. LB and RB, left board and right board of T‐DNA, respectively. (b) Schematic of the *OsPDS* target. The PAM is labelled in red, and the spacer sequence is highlighted in green. Blue arrows mark putative sites of DNA cleavage. (c) Cpf1‐induced *OsPDS* mutation in the T_0_ generation transgenic rice plants. WT, wild‐type sequence; ‐, deleted nucleotides; sequences in blue lowercase, insertions. The genotype of the mutation is indicated at the right of each sequence. ‐n, nucleotide deletion of the indicated number; ‐n/+n, simultaneous nucleotide deletion/insertion, respectively, of the indicated number at the site. (d) Phenotype of the *OsPDS* target mutated lines. From left to right, a nonmutated transgenic plant (*OsPDS*:*LbCpf1*‐pre‐crRNA type I line #2), a plant with a heterozygous mutation (*OsPDS*:*LbCpf1*‐pre‐crRNA type I line #9), a bi‐allelic mutant (*OsPDS*:*LbCpf1*‐pre‐crRNA type II line #8) and a putative null mutant with a chimeric mutation (*OsPDS*:*LbCpf1*‐pre‐crRNA type II line #19). Scale bar = 5 mm.

First, the coding sequence of a *Cpf1* ortholog from *Lachnospiraceae bacterium ND2006* (*LbCpf1;* NCBI accession number: NZ_JNKS01000011, gene locus_tag: T521_RS08385) was codon‐optimized for expression in rice, and a nuclear‐localization signal was attached to the 3′ ends (Data S1). To test whether crRNA‐Cpf1 could induce genome mutagenesis in plants, we selected a target sequence in an exon of the rice *phytoene desaturase* (*OsPDS*) gene as a spacer to design crRNAs through the fusion of direct repeat sequences and constructed it into an engineered binary vector (Figures 1a and b and S1). After the transgenic plants were generated, the target region was examined using the T7 endonuclease (T7E1) assay (Figure S2a) and Sanger sequencing (Figure S3), and the results revealed that targeted mutagenesis could be achieved (Figure 1c and Table 1). Two putative null mutants were identified (Figure 1c) and, as expected, both exhibited an albino phenotype (Figure 1d). Another target located in the rice *Bentazon‐sensitive‐lethal* (*OsBEL*) gene was used to further confirm the genome editing of Cpf1 (Figures S2b and 4). Consistent with the *OsPDS* target, the mutants were efficiently induced through the transformation of *LbCpf1* and its cognate crRNAs (Table 1). Cpf1 processes precursor crRNAs (pre‐crRNAs) into mature crRNAs to guide the endonuclease (Fonfara *et al*., 2016). Most CRISPR‐Cpf1 studies in mammalian cells have taken advantage of mature (processed) crRNAs (Hur *et al*., 2016; Kim *et al*., 2016; Zetsche *et al*., 2015). However, no mutant was identified from the transgenic plants generated by targeting either *OsPDS* or *OsBEL* (Table 1) directly using the mature crRNA sequence, suggesting that the mutagenesis efficiencies of mature crRNA‐Cpf1 complexes were low in the plants. By contrast, mutants were more frequently identified in the lines transformed with pre‐crRNAs (Table 1). We found that 13.6% and 20% of the transgenic plants targeting *OsPDS* and *OsBEL*, respectively, were mutated using pre‐crRNAs with the full‐length repeat‐spacer sequence (pre‐crRNA type I). Moreover, mutation frequencies of 21.4% and 41.2% were generated at the *OsPDS* and *OsBEL* targets, respectively, using longer pre‐crRNAs with the full‐length repeat‐spacer‐repeat sequence (pre‐crRNA type II). The higher mutation frequency of pre‐crRNAs in rice is consistent with the *in vitro* observation that the pre‐crRNA‐Cpf1 complexes exhibit stronger binding activity and increased nuclease activity than do mature crRNA‐Cpf1 (Fonfara *et al*., 2016).

**Table 1:** Targeted mutagenesis frequency of CRISPR‐Cpf1 in T_0_ transgenic plants

Genome target	crRNA type^a^	No. of events examined^b^	No. of events with mutation^b^	Mutation rate (%)
*OsPDS*	Mature crRNA	85	0	0
	Pre‐crRNA type I	22	3	13.6
	Pre‐crRNA type II	28	6	21.4
*OsBEL*	Mature crRNA	48	0	0
	Pre‐crRNA type I	20	4	20
	Pre‐crRNA type II	34	14	41.2

a The details of the crRNA types are given in Figures 1a and S1.

b Independent T_0_ events were used to examine the target mutation.

We identified several differences between the behaviour of Cpf1‐mediated mutagenesis and that of the previously reported Cas9 system. Compared with the common short indels (1~2 base pairs, bp) generated by Cas9 in rice (Zhang *et al*., 2014), most of the mutations generated by Cpf1 were relatively long (Figures 1c and S4), which might be caused by nonhomologous end‐joining repair on the 4~5‐nt 5′ overhangs resulting from the staggered cutting of Cpf1. In addition, several reports have indicated that homozygous mutations can be easily generated by CRISPR‐Cas9 in T_0_ plants (Shan *et al*., 2013; Zhang *et al*., 2014), which likely resulted from the Cas9‐induced DNA cleavage in the first cell. However, homozygous mutated lines generated by Cpf1 were not observed; instead, the mutated lines were either chimeric or heterozygous (Table 2). Because a heterozygous genotype could be generated by mutation in only a portion of cells, the results suggest that the Cpf1‐induced mutants might be largely somatic. Similar mosaicism has been frequently observed in Cpf1‐mutated mice (Kim *et al*., 2016), suggesting that Cpf1 should induce DNA cleavage after the first division of the embryogenic cell and might require a longer acting period than Cas9 *in vivo*.

**Table 2:** Zygosity in T_0_ rice mutants

Genome target	crRNA type	No. of mutants	Zygosity (%)^a^			
			Ho	Bi‐al	He	Ch
*OsPDS*	Pre‐crRNA type I	3	0	0	2(66.7)	1(33.3)
	Pre‐crRNA type II	6	0	1(16.7)	2(33.3)	3(50)
*OsBEL*	Pre‐crRNA type I	4	0	0	2(50)	2(50)
	Pre‐crRNA type II	14	0	2(14.3)	4(28.6)	8(57.1)

a Plant zygosity is considered homozygous (Ho) or bi‐allelic (Bi‐al) if the two copies of the target are mutated to the same or different types, respectively. A plant carrying both WT and mutated copies of the target is recognized as heterozygous (He). If more than two genotypes are found in the target, the zygosity is believed to be chimeric (Ch). The zygosity of the T_0_ plant is putative. The percentage in brackets indicates the frequency of the corresponding zygosity.

The off‐target effect of CRISPR‐Cpf1 might be minimal compared with that of CRISPR‐Cas9 in mammalian cells (Hur *et al*., 2016; Kim *et al*., 2016; Kleinstiver *et al*., 2016). To determine the off‐target effect of Cpf1 in plants, we selected genome sites with a high sequence similarity to the *OsPDS* or *OsBEL* targets (Table S1). For each target, five transgenic plants with on‐target mutations were separately analysed by direct Sanger sequencing of the potential off‐target sites. After examining the homologous sites with fewer than 7‐nt mismatches, we did not detect off‐target mutations. These results further support the finding that the CRISPR‐Cpf1 system, with careful target selection, is highly specific *in vivo*.

To determine whether the Cpf1‐generated mutation could be transmitted through the germ‐line, we analysed the T_1_ generation populations of several mutated T_0_ lines and found that the majority of the progeny were still mutants (Table 3). Furthermore, most of the T_0_ mutation types were identified in T_1_ plants, suggesting that CRISPR‐Cpf1‐mediated genome mutagenesis is heritable. Fragment‐specific PCR showed that the T‐DNA insertion could be segregated out in certain T_1_ mutants (Fig. S5), suggesting that similar to the CRISPR‐Cas9 system, CRISPR‐Cpf1 could rapidly generate transgene‐free, genome‐modified crops (Zhang *et al*., 2014).

**Table 3:** Transmission of Cpf1‐induced mutations from T_0_ transgenic rice to the T_1_ generation

Lines	T_0_ generation^a^		T_1_ generation		
	Mutation types (bp)	Zygosity	Mutation types (bp)	Mutant No./Test No. (%)^b^	T‐DNA segregation^c^
*OsPDS*:*LbCpf1*‐crRNA type I #3	−7	He	−7, −5	9/12 (75%)	10+:2−
*OsPDS*:*LbCpf1*‐crRNA type I #9	−28/+6	He	−28/+6, −7	10/12 (83.3%)	8+:4−
*OsPDS*:*LbCpf1*‐crRNA type I #17	−10, −32	He	−10, −32	12/12 (100%)	12+

a The genotype of each line is also indicated in Fig. 1c. The zygosity of the T_0_ plant is putative.

b A total of 12 T_1_ plants of each T_0_ mutant line were used to examine the targeted mutation. The frequency in the bracket was calculated using the number of T_1_ mutants as the numerator.

c +, number of T_1_ plants in which T‐DNA regions could be detected; −, number of T_1_ plants in which T‐DNA regions could not be detected.

The mouse was the first organism to be mutated with the CRISPR‐Cpf1 system. The mutants were obtained by transiently introducing a crRNA‐Cpf1 protein complex or a crRNA‐*Cpf1* mRNA mixture (Hur *et al*., 2016; Kim *et al*., 2016). The data obtained in this study show that the expression of CRISPR‐Cpf1 through stable transformation of the engineered vector can generate heritable targeted modifications in the rice genome. We believe that in plant species with reliable genetic transformation systems, the stable expression of CRISPR‐Cpf1 should efficiently produce mutants more rapidly and with a lower cost compared with the transient strategy and that this technique constitutes a robust and ready‐to‐use approach for plant genome mutagenesis. Due to the substantial differences between Cpf1 and Cas9 in terms of target recognition and DNA cleavage, CRISPR‐Cpf1 not only provides a new and alternative method for plant targeted mutagenesis but also greatly enhances the scope and precision of crop genome editing.

## Experimental procedures

### Vector construction

The coding sequence of *LbCpf1* was codon‐optimized for rice and synthesized by GeneWiz (Suzhou). The CRISPR‐Cpf1 binary vector was developed from the pHSN400 plasmid (Xing *et al*., 2014). The *Ubi* promoter, rice codon‐optimized *Cpf1* and 35S terminator (35‐T) were used instead of the 35S promoter, *Cas9* and Nos terminator in pHSN400 through *Hind*III/*Not*I, *Not*I/*Sac*I and *Sac*I/*Eco*RI double digestions, respectively. A cassette for OsU3‐driven crRNA expression was synthesized (Data S1) and inserted into the plasmid by *Hind*III digestion to construct the CRISPR‐Cpf1 binary vector.

To target the specific genome sites of *OsPDS* and *OsBEL*, we used 25‐ to 31‐nt sequences with the PAM sequence at their 5′‐ends as spacers. The spacers were fused with different repeat sequences to construct crRNAs (Figure S1). In accordance with a previous protocol (Xing *et al*., 2014), the double‐stranded crRNAs were assembled from two complementary oligos (Table S2) and inserted into the vector by *Bsa*I digestion. The colonies were positively selected using kanamycin and negatively selected using spectinomycin and then further confirmed by sequencing.

### Rice transformation and plant growth

The vectors were introduced into a pSOUP‐containing agrobacterium strain EH105 (Hellens *et al*., 2000). For each vector, 600 (for the mature crRNA vector) or 300 (for the pre‐crRNA vector) embryonic calluses of rice (*Oryza sativa* L. ssp. *japonica* cv. Nipponbare) were infected with the agrobacterium according to a previously described protocol (Duan *et al*., 2012). The transgenic plants were selected and regenerated under selection with 50 mg/L hygromycin. After two to three weeks of rooting, the T_0_ plants were transplanted into soil and grown in a glasshouse at 30 °C. The progeny were generated through strict self‐pollination of each individual T_0_ event.

### Genotyping of targeted mutations

The genomic DNA from the leaves of rice plants was isolated using the CTAB method. The genomic region containing the target site was amplified in the corresponding transgenic plants using site‐specific primers (Table S2) and the high‐fidelity HiFi PCR mixture (TransGen Biotech, Beijing). To detect the mutation, we mixed the PCR product of each transgenic plant with the product generated from the wild‐type plant and then analysed it using the T7E1 assay following a standard protocol (New England Biolabs, MA, USA). Moreover, the PCR products were sequenced directly using the respective forward primer or after cloning into a pEASY‐T vector (TransGen Biotech, Beijing). For each putative mutant event, at least 10 colonies were Sanger sequenced. Specific primers for the *LbCpf1*, crRNAs and *hygromycin phosphotransferase* (*HPT*) cassette were also designed (Table S2) to detect the presence of the T‐DNA fragment.

### Off‐target effect detection

The potential off‐target sites were predicted using the online tool Cas‐OFFinder (Bae *et al*., 2014). The sites of each target in five randomly selected mutant lines were examined by site‐specific PCR and direct Sanger sequencing. The sequences of the sites and related PCR primers are listed in Tables S1 and S3.

## Supporting information


**Figure S1** Design of different crRNA variants for Cpf1‐induced plant genome targeting.
**Figure S2** T7E1 assay to detect transgenic plants carrying target mutations.
**Figure S3** Sequencing chromatograms of Cpf1‐induced mutations in the *OsPDS* target.
**Figure S4** Cpf1‐induced mutations in the *OsBEL* target in the T_0_ generation transgenic rice plants.
**Figure S5**. Segregation of the T‐DNA fragment in representative T_1_ mutants.
**Table S1** Potential off‐target sites of the Cpf1‐targeting *OsPDS* and *OsBEL*.
**Table S2** Primers used for vector construction and genotyping.
**Table S3** Primers used to detect off‐target effects in rice plants.
**Data S1** Sequence of the rice codon‐optimized *LbCpf1* and crRNA expression cassette.
