# Supplementary material for: Generation of targeted mutant rice using a CRISPR‐Cpf1 system
Source: Plant Biotechnol J. 2017 Feb 19;15(6):713–7. doi: 10.1111/pbi.12669 (PMC5425385; doi:10.1111/pbi.12669)
Supplement: Supplementary file 1 — Figure S1 Design of different crRNA variants for Cpf1‐induced plant genome targeting. Figure S2 T7E1 assay to detect transgenic plants carrying target mutations. Figure S3 Sequencing chromatograms of Cpf1‐induced mutations in the OsPDS target. Figure S4 Cpf1‐induced mutations in the OsBEL target in the T0 generation transgenic rice plants. Figure S5. Segregation of the T‐DNA fragment in representative T1 mutants. Table S1 Potential off‐target sites of the Cpf1‐targeting OsPDS and OsBEL. Table S2 Primers used for vector construction and genotyping. Table S3 Primers used to detect off‐target effects in rice plants. Data S1 Sequence of the rice codon‐optimized LbCpf1 and crRNA expression cassette. [file PBI-15-713-s001.docx]

**Supporting information**

**Data S1.** Sequence of the rice codon-optimized *LbCpf1* and crRNA expression cassette.

**Supplementary Figure 1.** Design of different crRNA variants for Cpf1-induced plant genome targeting.

**Supplementary Figure 2.** T7E1 assay to detect transgenic plants carrying target mutations.

**Supplementary Figure 3.** Sequencing chromatograms of Cpf1-induced mutations in the *OsPDS* target.

**Supplementary Figure 4.** Cpf1-induced mutations in the *OsBEL* target in the T_0_ generation transgenic rice plants.

**Supplementary Figure 5.** Segregation of the T-DNA fragment in representative T_1_ mutants.

**Supplementary Table 1.** Potential off-target sites of the Cpf1-targeting *OsPDS* and *OsBEL.*

**Supplementary Table 2.** Primers used for vector construction and genotyping.

**Supplementary Table 3.** Primers used to detect off-target effects in rice plants.

**Data S1**

> *Os-LbCpf1*

(rice codon-optimized *LbCpf1*, 3’ end located NLS-3xHA is marked in orange)

ATGTCCAAGCTGGAGAAGTTTACAAACTGTTACAGCCTCTCCAAAACCCTCAGGTTTAAAGCGATCCCGGTGGGCAAGACCCAGGAGAACATCGACAACAAGAGGCTCCTGGTGGAAGACGAGAAGCGCGCCGAAGACTACAAGGGCGTGAAGAAGCTGCTCGATAGGTACTACCTCAGCTTTATTAACGACGTGCTGCACAGCATCAAACTCAAGAATCTCAACAACTACATCTCCCTCTTCCGCAAAAAGACCCGCACCGAGAAGGAGAACAAGGAGCTGGAGAACCTGGAGATCAACCTCCGCAAGGAAATCGCCAAAGCGTTCAAGGGCAATGAAGGGTACAAGAGCCTCTTCAAGAAAGACATCATCGAAACTATCCTCCCAGAGTTTCTCGATGACAAGGACGAGATCGCGCTGGTGAACTCCTTTAACGGGTTCACAACCGCGTTTACCGGCTTCTTTGATAACAGGGAAAATATGTTCTCCGAGGAGGCCAAGTCCACCAGCATCGCCTTCAGGTGTATCAACGAGAACCTCACCCGCTACATTTCCAATATGGACATTTTCGAGAAGGTGGATGCGATCTTCGATAAGCACGAGGTGCAGGAGATCAAAGAGAAGATTCTCAATTCCGATTATGACGTCGAGGATTTCTTCGAAGGGGAGTTCTTTAATTTTGTGCTCACACAAGAGGGCATTGACGTGTACAACGCGATTATCGGGGGCTTCGTCACAGAGTCCGGGGAGAAGATTAAGGGGCTGAATGAGTACATCAATCTGTACAATCAGAAGACCAAGCAGAAACTGCCGAAATTCAAGCCGCTCTACAAGCAAGTCCTGTCCGATAGGGAAAGCCTCTCCTTCTACGGCGAGGGCTATACCAGCGACGAGGAGGTGCTGGAAGTCTTCCGCAACACACTGAATAAGAATAGCGAGATTTTCTCCTCCATCAAGAAGCTCGAGAAGCTCTTTAAGAACTTTGACGAGTACAGCTCCGCCGGGATTTTCGTGAAGAACGGGCCGGCGATCAGCACCATCTCCAAGGACATCTTTGGCGAGTGGAACGTCATCAGGGACAAGTGGAACGCCGAGTACGACGACATCCACCTGAAGAAGAAGGCGGTGGTGACCGAGAAGTATGAGGACGATCGCAGGAAGTCCTTCAAAAAAATCGGCTCCTTCAGCCTCGAACAGCTCCAGGAGTATGCCGATGCGGATCTGTCCGTCGTCGAGAAGCTGAAGGAAATCATCATTCAGAAGGTCGACGAGATCTATAAAGTGTACGGGTCCAGCGAGAAGCTGTTCGACGCCGACTTTGTGCTCGAGAAGTCCCTCAAAAAGAATGACGCCGTGGTGGCCATTATGAAAGACCTGCTCGACTCCGTGAAGTCCTTCGAAAATTACATTAAAGCGTTCTTTGGGGAGGGGAAGGAAACTAACAGGGATGAGTCCTTCTATGGCGACTTTGTCCTCGCGTACGACATCCTGCTGAAGGTCGACCACATTTACGACGCGATCCGCAACTACGTGACACAGAAGCCGTACTCCAAAGACAAGTTCAAGCTGTACTTCCAGAACCCGCAATTTATGGGGGGCTGGGACAAGGATAAAGAGACAGACTACCGCGCGACAATTCTCCGCTATGGCTCCAAATACTATCTGGCCATCATGGACAAGAAGTACGCGAAGTGCCTGCAGAAGATCGACAAAGACGACGTCAATGGCAACTATGAAAAGATCAACTACAAGCTGCTGCCGGGCCCGAACAAGATGCTCCCGAAGGTGTTCTTCAGCAAGAAGTGGATGGCCTACTACAATCCAAGCGAGGATATTCAGAAAATCTATAAAAACGGGACCTTCAAGAAGGGGGACATGTTTAACCTCAACGACTGCCACAAGCTCATTGATTTCTTCAAGGATAGCATTTCCCGCTACCCGAAATGGTCCAATGCGTACGATTTTAACTTCTCCGAGACAGAAAAGTACAAAGACATCGCGGGCTTTTACAGGGAGGTGGAGGAGCAAGGGTATAAAGTTTCTTTTGAATCCGCGAGCAAGAAGGAAGTCGACAAGCTCGTCGAGGAGGGCAAGCTCTACATGTTCCAAATTTATAACAAGGACTTTTCCGACAAGAGCCATGGGACCCCAAACCTCCACACCATGTACTTCAAACTGCTCTTTGACGAGAACAACCACGGGCAAATCAGGCTGAGCGGCGGCGCCGAATTATTCATGCGCAGGGCCTCCCTCAAGAAGGAAGAGCTGGTCGTCCATCCAGCCAATTCCCCGATCGCGAACAAGAACCCGGACAATCCGAAAAAGACCACCACCCTGTCCTACGACGTCTACAAGGACAAACGCTTCAGCGAAGACCAGTACGAATTACACATCCCAATTGCGATTAATAAGTGCCCAAAGAATATCTTCAAAATTAATACAGAGGTCAGGGTGCTGCTCAAACACGACGACAATCCGTATGTCATCGGCATTGACAGGGGCGAGCGCAATCTGCTCTATATCGTGGTCGTGGATGGGAAGGGCAATATTGTGGAGCAGTACTCCCTGAACGAGATTATCAACAACTTCAATGGGATTAGGATTAAGACCGACTATCACAGCCTGCTCGACAAGAAAGAAAAAGAGAGGTTTGAGGCCCGCCAAAACTGGACCTCCATTGAGAATATCAAAGAATTAAAGGCCGGCTATATTTCCCAAGTCGTCCACAAGATCTGCGAGCTGGTGGAGAAATATGACGCCGTGATTGCGCTCGAAGACTTAAATTCTGGGTTCAAGAACTCCCGCGTGAAGGTGGAAAAACAGGTGTATCAGAAATTCGAGAAAATGCTGATCGACAAACTCAATTATATGGTGGATAAGAAGTCCAACCCGTGTGCCACAGGGGGCGCGCTGAAGGGCTATCAGATCACCAACAAGTTCGAGAGCTTCAAGAGCATGAGCACCCAGAACGGGTTTATTTTCTACATCCCGGCGTGGCTCACCTCCAAGATTGACCCGAGCACCGGCTTCGTGAACCTCCTGAAGACAAAGTATACCTCCATTGCCGACAGCAAGAAGTTTATCTCCTCCTTCGACCGCATTATGTATGTGCCGGAGGAGGACCTCTTCGAGTTCGCCCTCGACTACAAAAACTTCAGCCGCACAGATGCGGATTACATCAAGAAGTGGAAGCTGTACTCCTACGGGAACAGGATCCGCATCTTCAGGAATCCAAAAAAAAATAACGTCTTTGACTGGGAGGAAGTGTGCCTGACATCCGCCTACAAGGAACTGTTCAATAAATACGGCATCAATTACCAGCAGGGCGACATTCGCGCCCTCCTCTGTGAGCAGTCCGACAAAGCGTTTTACTCCAGCTTCATGGCCCTCATGTCCCTGATGCTCCAAATGAGGAATAGCATCACAGGGCGCACCGACGTCGACTTCCTCATCAGCCCGGTGAAGAACTCCGACGGGATCTTTTACGACTCCCGCAACTATGAGGCGCAAGAGAATGCGATCCTCCCGAAGAACGCCGATGCGAACGGGGCCTATAATATCGCCAGGAAAGTGCTCTGGGCCATCGGGCAGTTCAAAAAGGCGGAGGATGAGAAGCTCGACAAGGTGAAAATTGCCATTTCCAACAAGGAGTGGCTGGAGTACGCGCAGACCTCCGTGAAGCACAAAAGGCCGGCGGCCACGAAAAAGGCCGGCCAGGCAAAAAAGAAAAAGGGATCCTACCCATACGATGTTCCAGATTACGCTTATCCCTACGACGTGCCTGATTATGCATACCCATATGATGTCCCCGACTATGCCTAA

>OsU3-crRNA cassette

aagggatctttaaacatacgaacagatcacttaaagttcttctgaagcaacttaaagttatcaggcatgcatggatcttggaggaatcagatgtgcagtcagggaccatagcacaagacaggcgtcttctactggtgctaccagcaaatgctggaagccgggaacactgggtacgttggaaaccacgtgatgtgaagaagtaagataaactgtaggagaaaagcatttcgtagtgggccatgaagcctttcaggacatgtattgcagtatgggccggcccattacgcaattggacgacaacaaagactagtattagtaccacctcggctatccacatagatcaaagctgatttaaaagagttgtgcagatgatccgtggcaagagaccaacccagtggacataagcctgttcggttcgtaagctgtaatgcaagtagcgtatgcgctcacgcaactggtccagaaccttgaccgaacgcagcggtggtaacggcgcagtggcggttttcatggcttgttatgactgtttttttggggtacagtctatgcctcgggcatccaagcagcaagcgcgttacgccgtgggtcgatgtttgatgttatggagcagcaacgatgttacgcagcagggcagtcgccctaaaacaaagttaaacatcatgggggaagcggtgatcgccgaagtatcgactcaactatcagaggtagttggcgtcatcgagcgccatctcgaaccgacgttgctggccgtacatttgtacggctccgcagtggatggcggcctgaagccacacagtgatattgatttgctggttacggtgaccgtaaggcttgatgaaacaacgcggcgagctttgatcaacgaccttttggaaacttcggcttcccctggagagagcgagattctccgcgctgtagaagtcaccattgttgtgcacgacgacatcattccgtggcgttatccagctaagcgcgaactgcaatttggagaatggcagcgcaatgacattcttgcaggtatcttcgagccagccacgatcgacattgatctggctatcttgctgacaaaagcaagagaacatagcgttgccttggtaggtccagcggcggaggaactctttgatccggttcctgaacaggatctatttgaggcgctaaatgaaaccttaacgctatggaactcgccgcccgactgggctggcgatgagcgaaatgtagtgcttacgttgtcccgcatttggtacagcgcagtaaccggcaaaatcgcgccgaaggatgtcgctgccgactgggcaatggagcgcctgccggcccagtatcagcccgtcatacttgaagctagacaggcttatcttggacaagaagaagatcgcttggcctcgcgcgcagatcagttggaagaatttgtccactacgtgaaaggcgagatcaccaaggtagtcggcaaataatgtctagctagaaattcgttcaagccgacgccgcttcgcggcgcggcttaactcaagcgttagatgcactaagcacataattgctcacagccaaactatcaggtcaagtctgcttttattatttttaagcgtgcataataagccggtctcattttttttagtagtagcatctgac

crRNA insertion:

5’-GGCANNN….NNN-3’

3’-NNN….NNNAAAA-3’

Note: The OsU3 promoter is marked in blue, and the poly-T terminator is marked in red. Two *Bsa*I sites (underlined) and the spectinomycin resistance marker SpR (marked in orange) were used for crRNA insertion and construct selection.

**Supplementary Figure 1**

**
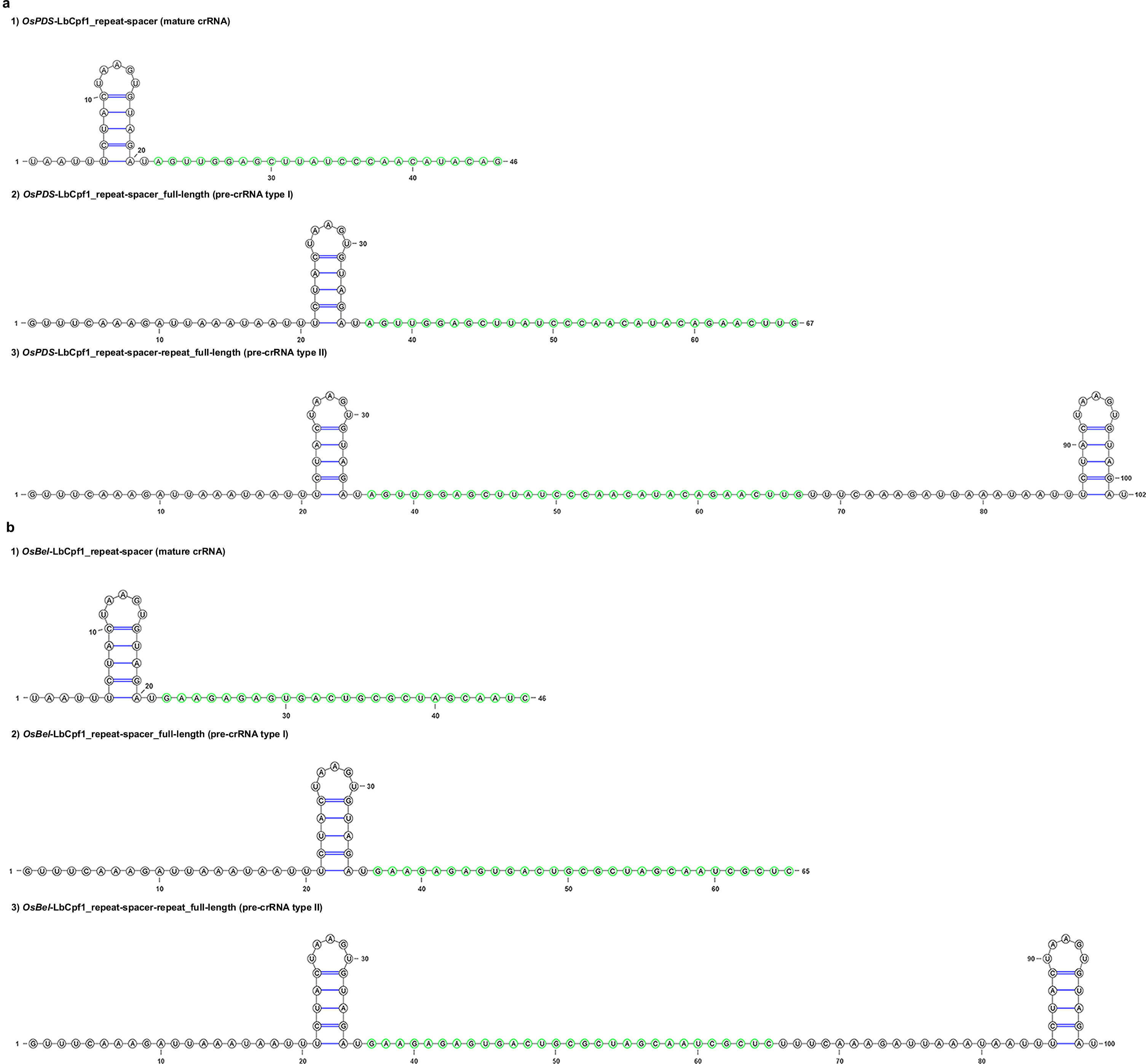
**

**Supplementary Figure 1.** Design of different crRNA variants for Cpf1-induced plant genome targeting.

Three types of crRNAs were designed to induce mutations in the *OsPDS* (a) and *OsBEL* targets (b). The spacer and repeat regions are labeled in green and black, respectively. For each specific spacer, a mature crRNA fused with a short (processed) repeat sequence, a pre-crRNA type I with a full-length repeat sequence and a pre-crRNA type II with two full-length repeat sequences on both ends of the spacer were synthesized to test the mutation frequency in the corresponding transgenic plants.

**Supplementary Figure 2**

**
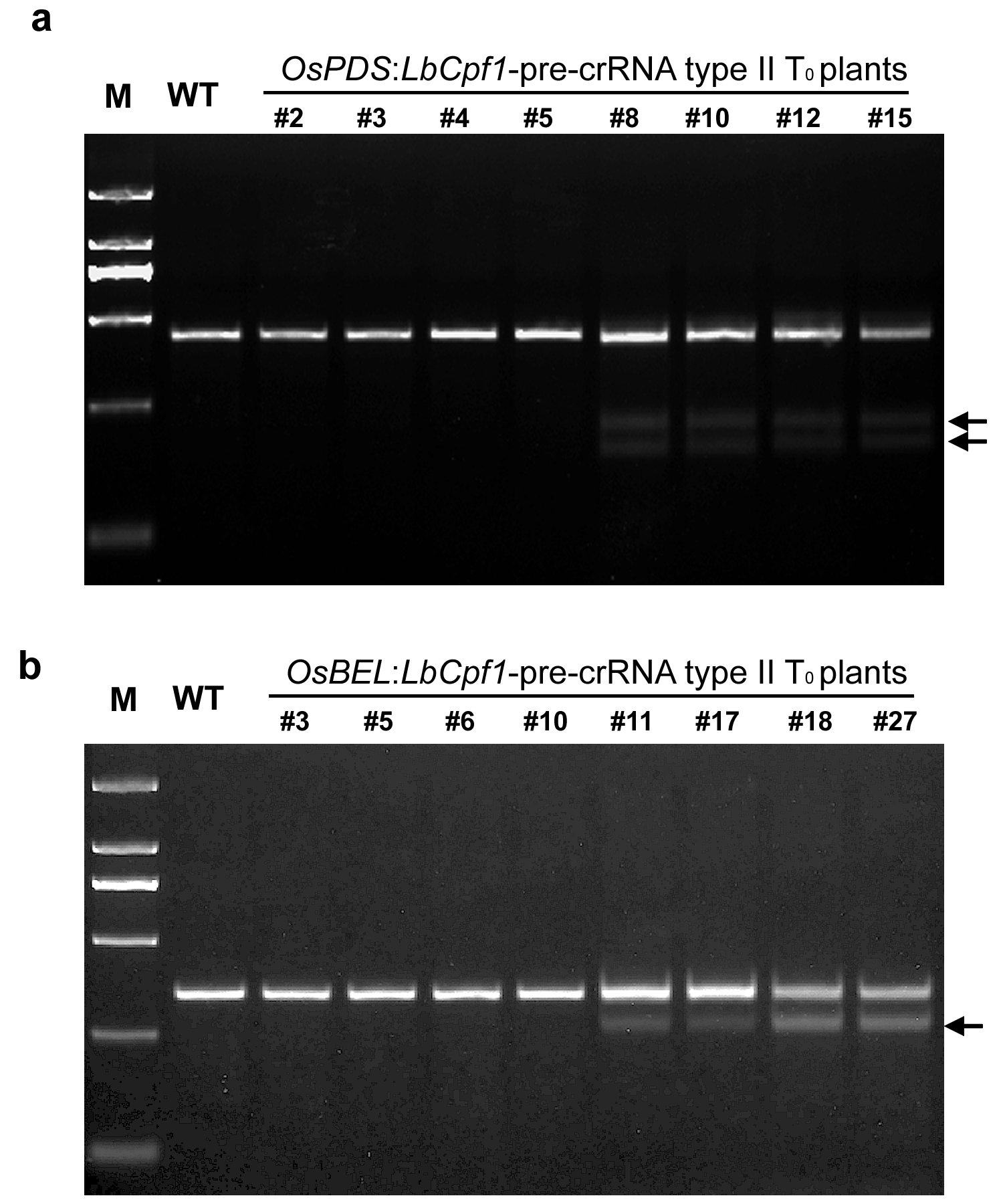
**

**Supplementary Figure 2.** T7E1 assay to detect transgenic plants carrying target mutations.

From left to right: M, marker; WT, wild-type plant; four transgenic lines not carrying mutations and four representative lines carrying mutations in the *OsPDS* target (a) or the *OsBEL* target (b). The arrows indicate predicted bands cut by T7 endonuclease.

**Supplementary Figure 3.**

**
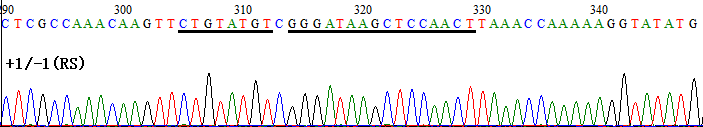
**


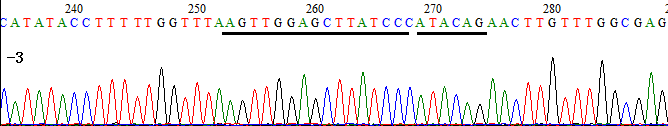


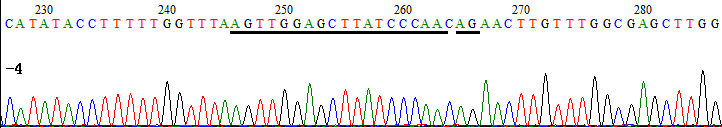


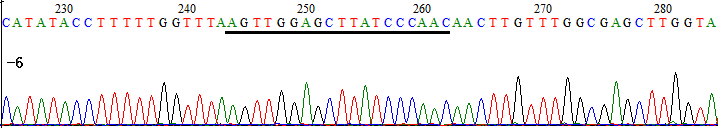


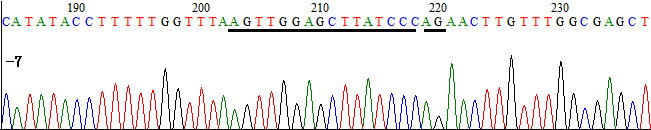


**
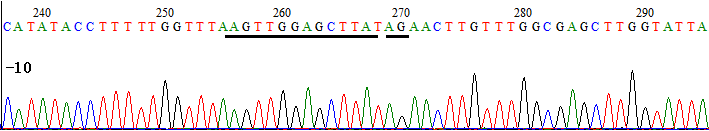
**


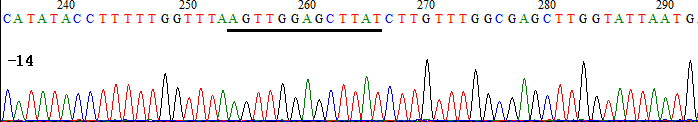


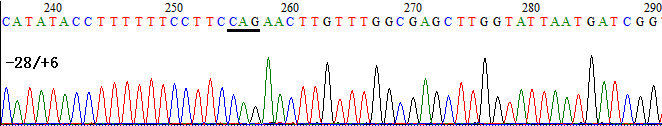


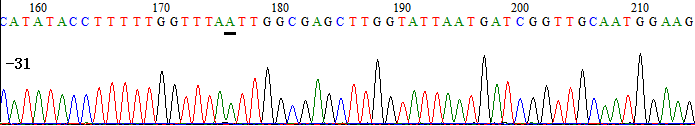


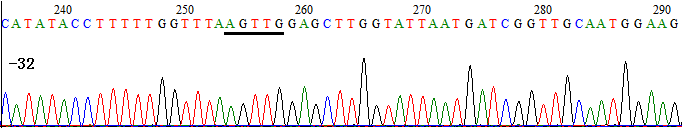


**Supplementary Figure 3.** Sequencing chromatograms of Cpf1-induced mutations in the *OsPDS* target.

The mutation types are indicated on the left. The target sequence is underlined. RS, reverse strand.


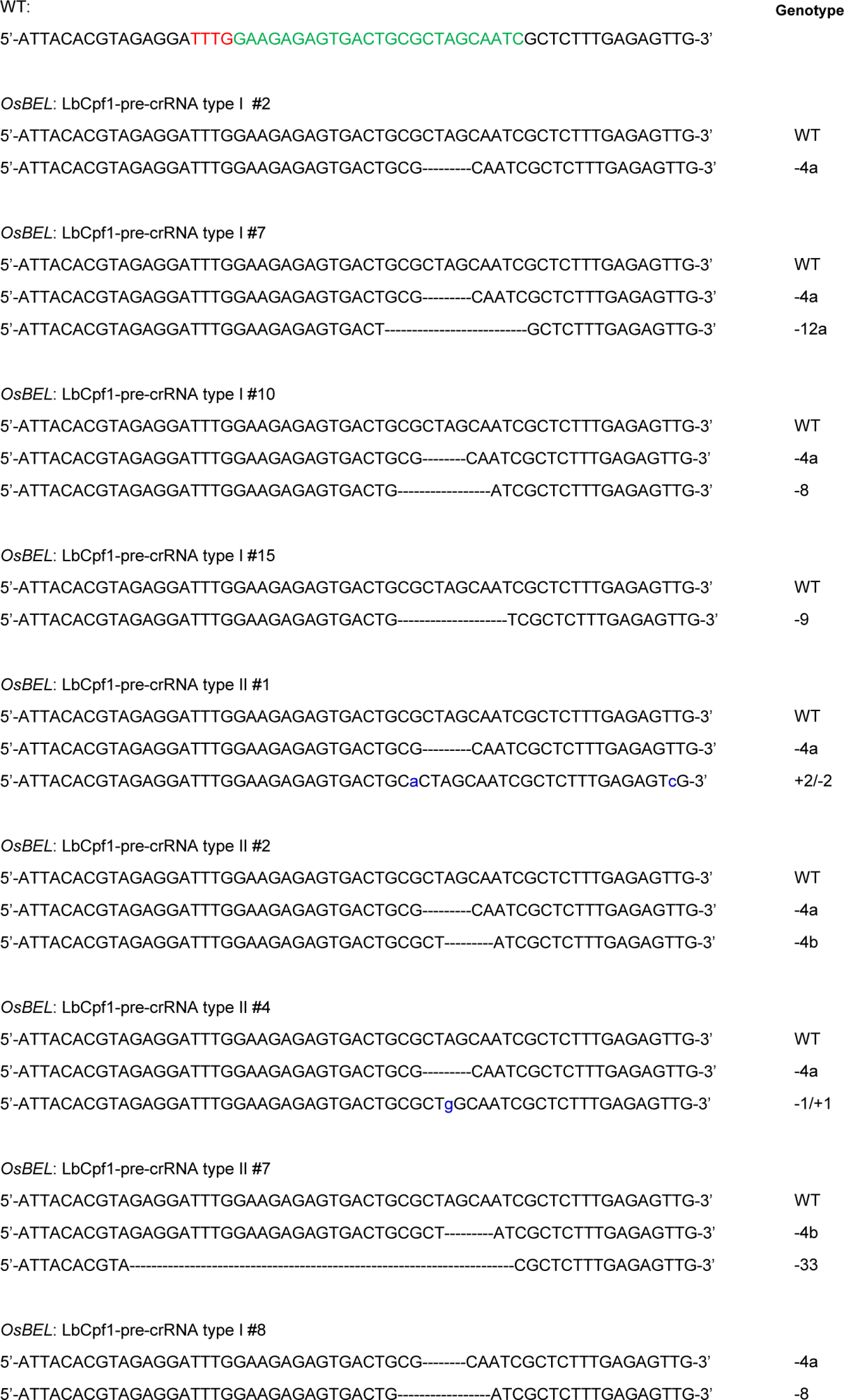
**Supplementary Figure 4**

**a.**

**
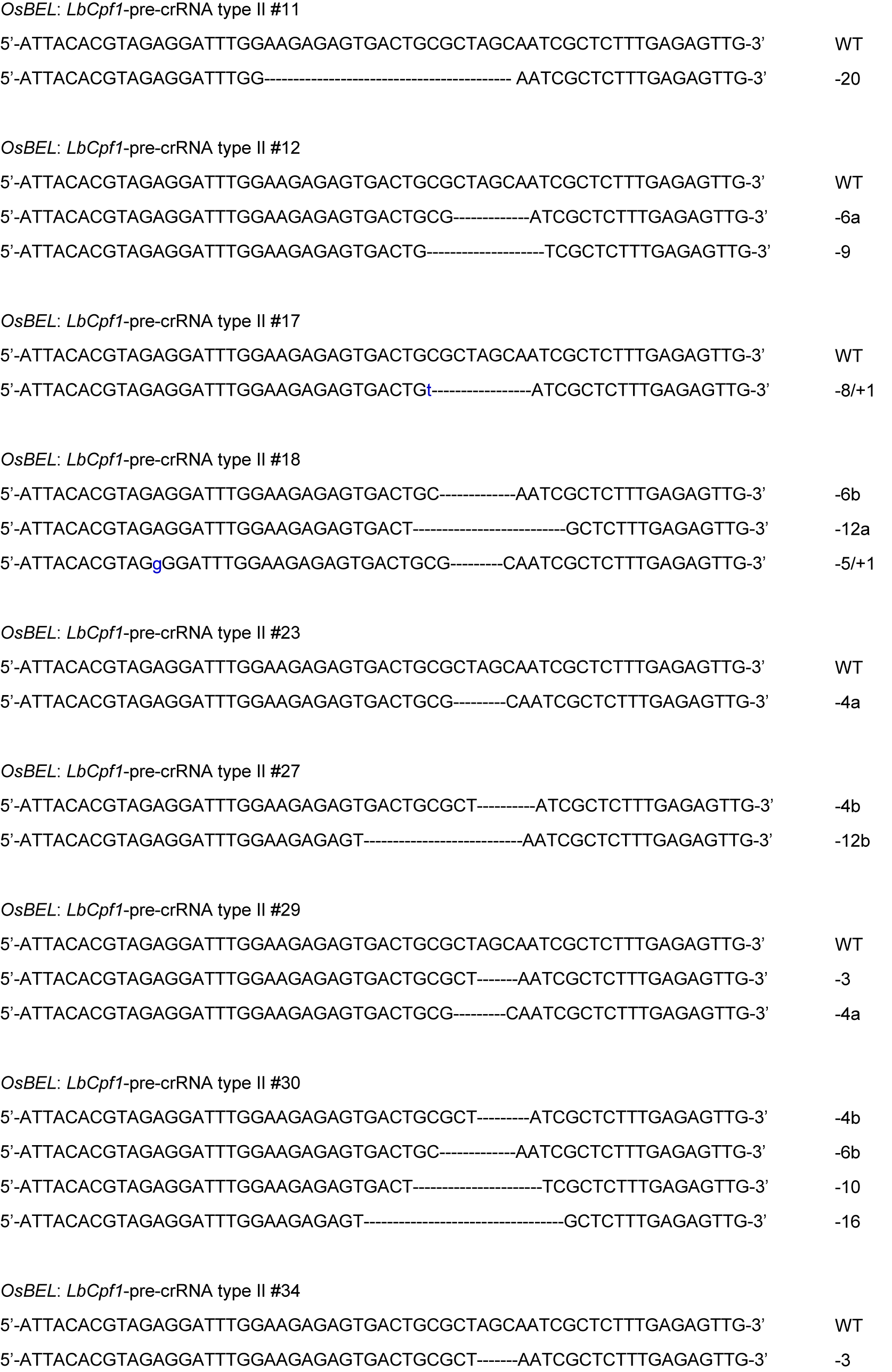
**

**b.**


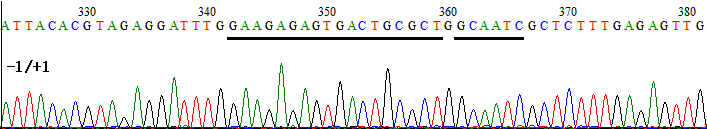


**
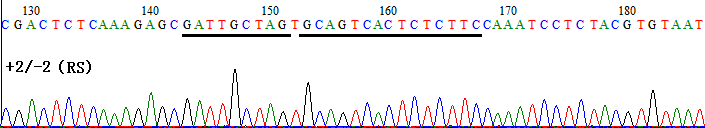
**


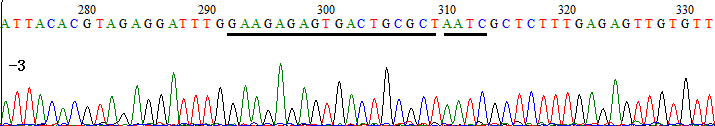


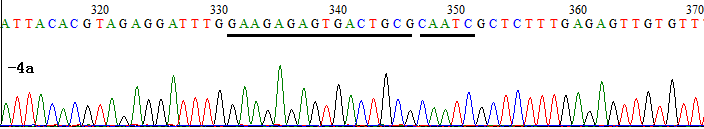


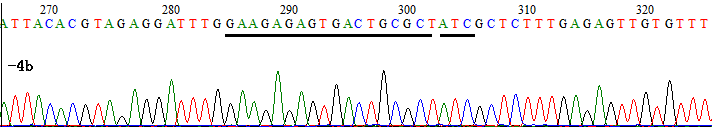


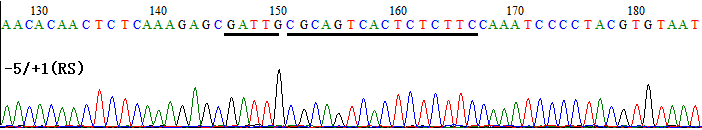


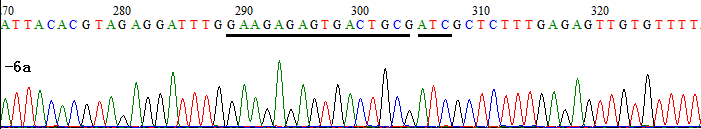


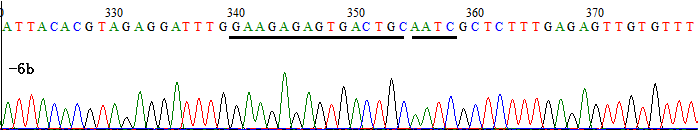


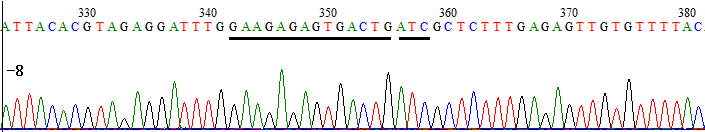


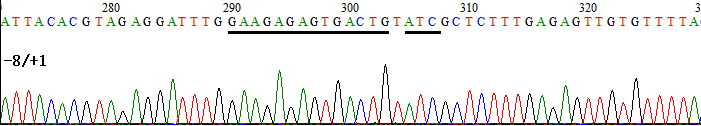


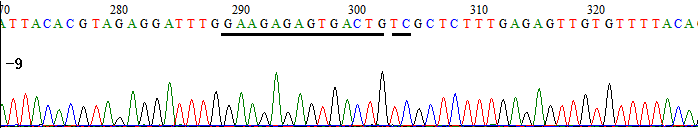


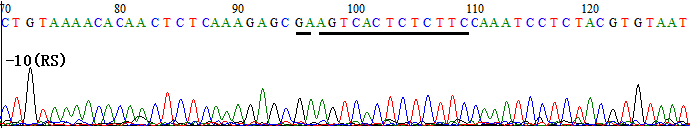


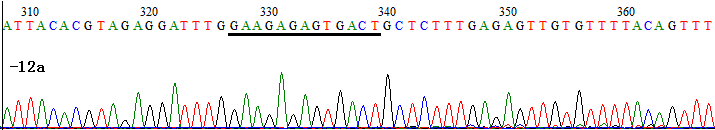


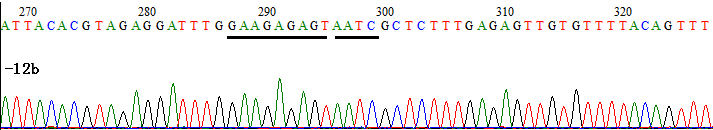


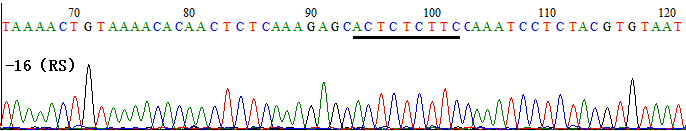


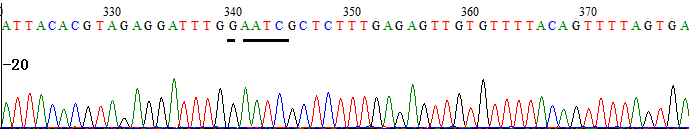


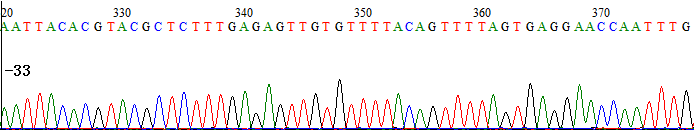


**Supplementary Figure 4.** Cpf1-induced mutations in the *OsBEL* target in the T_0_ generation transgenic rice plants.

a. Sequence alignment of the target regions. WT, wild-type sequence; -, deleted nucleotides; sequences in blue lowercase, insertions. The genotype of the mutation is indicated to the left of each sequence. -n, nucleotide deletion of the indicated number; -n/+n, simultaneous nucleotide deletion/insertion, respectively, of the indicated number at the site; -na/b, same number (n) deletions of different nucleotides. B. Sequencing chromatograms of exemplary mutations. The target sequence in the corresponding mutated allele is underlined.

**Supplementary Figure 5**

**
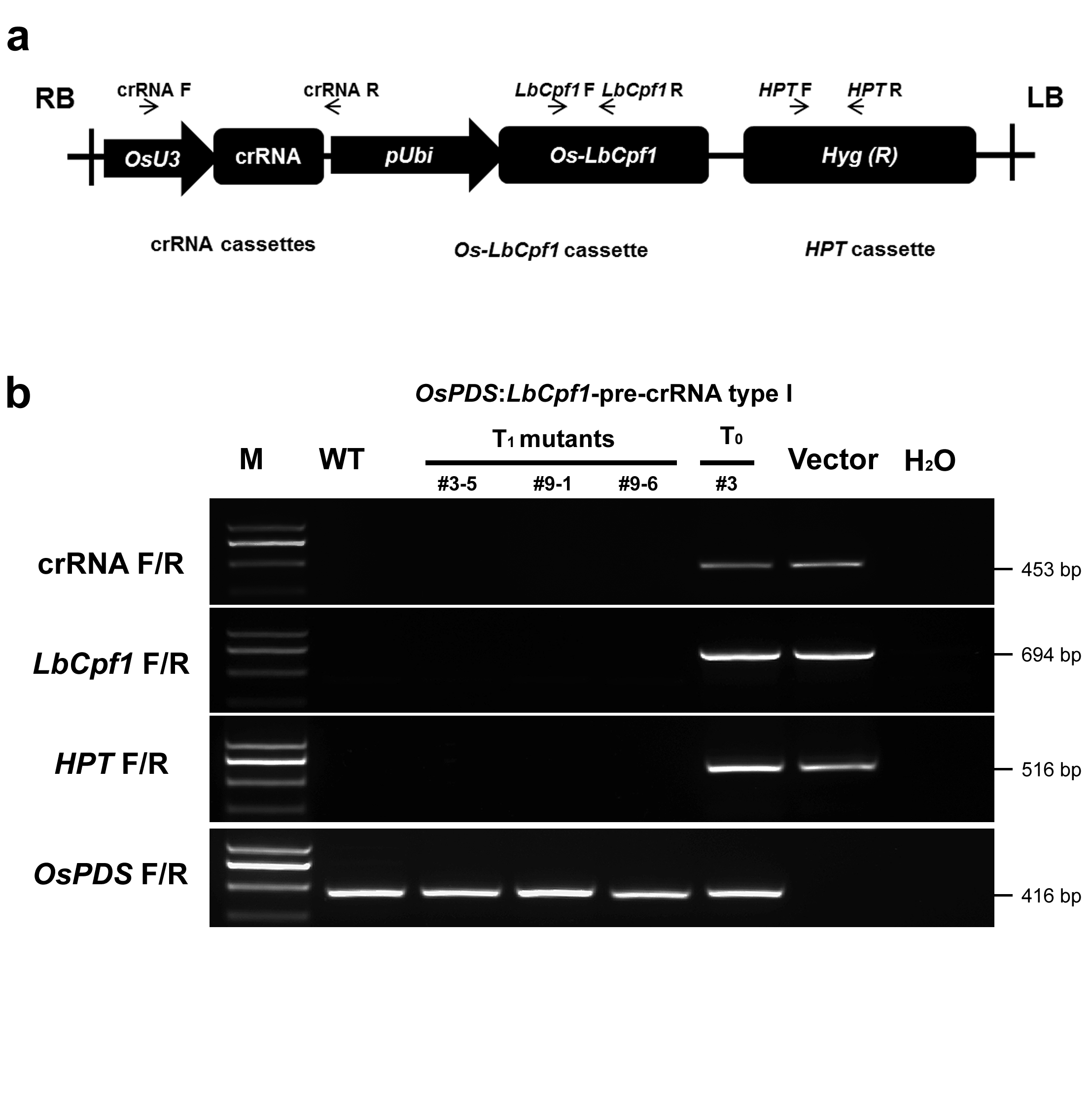
**

**Supplementary Figure 5.** Segregation of T-DNA fragments in representative T_1_ mutants.

Top, schematic of the T-DNA region showing the three pairs of site-specific primers used to detect the crRNA, *Cpf1* and *HPT* cassettes in plants. Bottom, detection of T-DNA in T_1_ mutants by PCR and gel electrophoresis. The T_0_ mutant and the corresponding CRISPR-Cpf1 vector were used as positive controls; the wild-type (WT) plant and H_2_O were used as negative controls. The target region of *OsPDS* was amplified by PCR as an internal control. M, DNA marker. The numbers on the right indicate the expected sizes of the PCR products.**Supplementary Table 1.** Potential off-target sites of the Cpf1-targeting *OsPDS* and *OsBEL.* Homologous sequences with a mismatch of up to 7 nt with the target sequence were selected and examined as off-target sites. The mismatches are labeled in red lowercase.

| **Target** | **Off-target No.** | **Sequence** | **Chr.** | **Position** | **Direction** | **Mismatches (nt)** |
| --- | --- | --- | --- | --- | --- | --- |
| *OsPDS* | On-Target | AGTTGGAGCTTATCCCAACATACAG | chr3 | 4390505 | + | 0 |
|  | OFF-1 | AaTTGGAaCTTAgCCCAtCAaACAG | chr7 | 20971438 | + | 5 |
|  | OFF-2 | AtTTGGAGCTTATtCgAAggTAaAG | chr8 | 15718880 | - | 6 |
|  | OFF-3 | caTTtGcGCTTATCCgAACATAtgG | chr3 | 720506 | + | 7 |
|  | OFF-4 | tGgTtGAGCTTAggCCAACcTACtG | chr8 | 3056254 | + | 7 |
|  | OFF-5 | AGTTtGAcCaTATCCCAAaAggaAG | chr8 | 3127137 | - | 7 |
|  | OFF-6 | AGTTtGgGCaTATaCCAAtATtgAG | chr8 | 7481032 | + | 7 |
|  | OFF-7 | AtTTGGAGCTTgTtCgAAggTAaAG | chr8 | 16025152 | - | 7 |
|  | OFF-8 | AGTTaGtGgTTtTCtCAACAaACAa | chr12 | 12377385 | - | 7 |
|  | OFF-9 | AtTTcaAGCTTgTCCCAAaATgCAa | chr12 | 19289915 | + | 7 |
|  | OFF-10 | AGTTGGAtCTaATCCtAgCATgtAa | chr12 | 25491111 | + | 7 |
| *OsBEL* | On-Target | GAAGAGAGTGACTGCGCTAGCAATC | chr3 | 31384860 | + | 0 |
|  | OFF-1 | AAGAGAacGACTGCtCTAGCAtTa | chr3 | 31378274 | + | 5 |
|  | OFF-2 | aAAGAGAGaGACTGCcaTcGCcATC | chr12 | 20493075 | + | 6 |
|  | OFF-3 | aAAGAGAGgGACTGCtCTctCcgTC | chr3 | 32557368 | - | 7 |
|  | OFF-4 | aAAGAaAGTGACaaaGCTAGCcATt | chr4 | 22526504 | - | 7 |
|  | OFF-5 | GAAacaAGTtACTGtGCTAGaAATa | chr5 | 13928143 | - | 7 |
|  | OFF-6 | cttGgGAGTGtgTGgGCTAGCAATC | chr5 | 27882035 | - | 7 |
|  | OFF-7 | GAgGAGAGaGACTaCtaTAGCAcaC | chr8 | 10300190 | - | 7 |
|  | OFF-8 | GAAaAGgtTcAtTtCGgTAGCAATC | chr8 | 23484691 | + | 7 |
|  | OFF-9 | GcAGAGAGTGAactCGtTAGCAtTt | chr12 | 3556465 | - | 7 |

**Supplementary Table 2.** Primers used for vector construction and genotyping.

| **1. Primers used to construct crRNAs** | |
| --- | --- |
| **crRNA** | **Oligos (5’ to 3’)** |
| *OsPDS:*  *LbCpf1*-mature crRNA | F: GGCATAATTTCTACTAAGTGTAGATAGTTGGAGCTTATCCCAACATACAG  R: AAAACTGTATGTTGGGATAAGCTCCAACTATCTACACTTAGTAGAAATTA |
| *OsPDS:*  *LbCpf1-*pre-crRNA type I | F: GGCAGTTTCAAAGATTAAATAATTTCTACTAAGTGTAGATAGTTGGAGCTTATCCCAACATACAGAACTTG  R: AAAACAAGTTCTGTATGTTGGGATAAGCTCCAACTATCTACACTTAGTAGAAATTATTTAATCTTTGAAAC |
| *OsPDS:*  *LbCpf1*-pre-crRNA type II | F: GGCAGTTTCAAAGATTAAATAATTTCTACTAAGTGTAGATAGTTGGAGCTTATCCCAACATACAGAACTTGTTTCAAAGATTAAATAATTTCTACTAAGTGTAGAT  R: AAAAATCTACACTTAGTAGAAATTATTTAATCTTTGAAACAAGTTCTGTATGTTGGGATAAGCTCCAACTATCTACACTTAGTAGAAATTATTTAATCTTTGAAAC |
| *OsBEL:*  *LbCpf1*-mature crRNA | F: GGCATAATTTCTACTAAGTGTAGATGAAGAGAGTGACTGCGCTAGCAATC  R: AAAAGATTGCTAGCGCAGTCACTCTCTTCATCTACACTTAGTAGAAATTA |
| *OsBEL:*  *LbCpf1-*pre-crRNA type I | F: GGCAGTTTCAAAGATTAAATAATTTCTACTAAGTGTAGATGAAGAGAGTGACTGCGCTAGCAATCGCTC  R: AAAAGAGCGATTGCTAGCGCAGTCACTCTCTTCATCTACACTTAGTAGAAATTATTTAATCTTTGAAAC |
| *OsBEL:*  *LbCpf1*-pre-crRNA type II | F: GGCAGTTTCAAAGATTAAATAATTTCTACTAAGTGTAGATGAAGAGAGTGACTGCGCTAGCAATCGCTCTTTCAAAGATTAAATAATTTCTACTAAGTGTAGAT  R: AAAAATCTACACTTAGTAGAAATTATTTAATCTTTGAAAGAGCGATTGCTAGCGCAGTCACTCTCTTCATCTACACTTAGTAGAAATTATTTAATCTTTGAAAC |

| **2. Primers used to assess on-target mutations** | |
| --- | --- |
| **Target** | **Oligos (5’ to 3’)** |
| *OsPDS* | FP: CCTCCTGTTCATACATAGTCTT  RP: TTAGTAAAACAAAAGTAACCAC |
| *OsBEL* | FP: GTGGAGGTCGACATGACTGAAG  RP: TTGCACATTCATACAAATTGGT |

| **3. Primers used to assess T-DNA insertions** | |
| --- | --- |
| **Target** | **Oligos (5’ to 3’)** |
| *LbCpf1* | FP: TTCACAACCGCGTTTACC  RP: TCACCACCGCCTTCTTCT |
| *crRNA* | FP: CGTGATGTGAAGAAGTAAGA  RP: GATAAACTGCACTTCAAACA |
| *HPT* | FP: ATCGTTATGTTTATCGGCACTTTG  RP: TGTTGGCGACCTCGTATTGG |

**Supplementary Table 3.** Primers used to detect off-target effects in rice plants.

| **Target** | **OFF-target No.** | **Forward primer (5' to 3')** | **Reverse primer (5' to 3')** | **Length of PCR product (bp)** |
| --- | --- | --- | --- | --- |
| *OsPDS* | OFF-1 | CTATCTCCGCCGATTTGTTC | GACGCGACGTGACATTCCAT | 509 |
|  | OFF-2 | AAATTGTAGGCAAGAAGAAAGT | GTCAGGAGTGGTAGAAGGAATG | 519 |
|  | OFF-3 | GGTAAGTCTTCCCTCCTGTG | CAATTCAATAACCTCCAACA | 657 |
|  | OFF-4 | TGGTGGAATGATTGGTGAGT | TAGGGAATGGTGTTGCTATG | 490 |
|  | OFF-5 | TATTAGTCATTGCACTTGTC | ATATGCTAAACTTACTTCGT | 449 |
|  | OFF-6 | TTGTCAAGTCGCCAGCAGTT | GTCCGTCCAGAGCAGGTTAG | 603 |
|  | OFF-7 | GGGTTAGGGTGTTGATAGCG | AGATGGGAGGGTGTCAGGAG | 603 |
|  | OFF-8 | AAAGATAAGTACAAATGTCCCACC | ATCGGCAGATTCGATTCACC | 525 |
|  | OFF-9 | GATCTAGCTCGGCAGGATAA | ATGCGGACAACAAACATCAC | 684 |
|  | OFF-10 | CAGTGGAATCCAATGCGTCTA | ACAATTAACCCGATCCCTTA | 448 |
| *OsBEL* | OFF-1 | CCAGTGCTTCGACTGGGAGAGG | GTTGACCGACTGACCGTATTAG | 420 |
|  | OFF-2 | ATCCAAGCAATCGGAGACACTA | GAAAGAAACATAGCGTAGCGAT | 466 |
|  | OFF-3 | CATATGTGCCTCTGGTTATTGG | TATCCGGTACTAGATTGATTTT | 582 |
|  | OFF-4 | TTTAGAAGGAAGACGGACGAGA | GAACTTTGTGGCATGGGAAGAG | 351 |
|  | OFF-5 | ATTTACAATTACAGGCTTCACC | AGATAAATGCGCTACTACATGA | 582 |
|  | OFF-6 | CGCACGTTATTACCACCTCGTT | GTCGACAATGGTGACGCTTTGG | 702 |
|  | OFF-7 | AGGTATCAAGGTGGAGGTTCCT | CCATATTCTCACGAGCTGAATT | 533 |
|  | OFF-8 | CCAGTTGAAATTCGACCTCCCT | AGGCCCACTTGTAAGCCAGAGA | 641 |
|  | OFF-9 | TGCCAACAACCAGAAATACCT | GCACTTTGCTTGATTCGGTAAT | 375 |
